# ASSOCIATION STUDY OF DNA METHYLATION AGE AND BONE HEALTH PHENOTYPES IN OLDER TWINS

**DOI:** 10.1093/geroni/igae098.2286

**Published:** 2024-12-31

**Authors:** Mette Soerensen, Kaare Christensen, Moustapha Kassem

**Affiliations:** University of Southern Denmark, Odense M, Syddanmark, Denmark; University of Southern Denmark, Odense M, Syddanmark, Denmark; University of Southern Denmark, Odense M, Syddanmark, Denmark

## Abstract

DNA methylation age, a biomarker for biological aging, is reported to associate to mortality and several aging-related phenotypes, while studies of bone phenotypes are surprisingly rare. Here we explore the association between diagnoses on age-related bone fractures, osteoporotic bone fractures, and osteoporosis without factures from the Danish Patient Registry to seven DNA methylation clocks: Horvath, IEAA, Hannum, EEAA PhenoAge, GrimAge and DunedinPACE. Cox regression analysis in a discovery sample of 310 older twins (age 73-91) revealed all clocks to reflect an increased epigenetic age with increasing risk of diagnosis; especially GrimAge and DunedinPACE appeared consistent across all three disease groups. A replication cohort of 777 twins (age 30-79) reflected the same direction of effect, with DunedinPACE displaying statistical significance. A twin pair analysis, reducing genetic and environmental confounding, confirmed the direction of effects, with GrimAge holding the largest effect sizes (e.g., HR=1.21, P=0.057 for osteoporotic fractures). In all analysis the DNA methylation-estimated telomere length reflected the opposite direction of effect, i.e., shorter telomere length with increased risk of diagnosis. Lastly, for a subset of the replication cohort (N=288, age 30-76) data on bone mineral density (BMD) and the bone turn over markers CTX and P1NP were available; CTX and P1NP showed decreased levels with increasing epigenetic age, while BMD showed an inconsistent pattern. Interaction analysis revealed age and sex effects for some of the clocks for BMD, perhaps pointing to the complex mechanisms behind bone biology. Taken together we report associations between DNA methylation age and bone health phenotypes.

